# Factors associated with retention in a smoking cessation trial for persons with a mental illness: a descriptive study

**DOI:** 10.1186/s12874-018-0640-5

**Published:** 2018-12-27

**Authors:** Alexandra P. Metse, Nur Ashikin Noor Hizam, John Wiggers, Paula Wye, Jenny A. Bowman

**Affiliations:** 1 Psychology administration office, University of Newcastle, University Drive, Callaghan, NSW 2308 Australia; 2 Hunter Medical Research Institute, Lot 1 Kookaburra Circuit, New Lambton Heights, NSW 2305 Australia; 3 Murdoch University, South Street, Murdoch, WA 6150 Australia; 4 Hunter New England Population Health, Longworth Ave, Wallsend, NSW 2287 Australia

**Keywords:** Smoking, Mental illness, Retention, Attrition, Mental health services

## Abstract

**Background:**

Exploring factors associated with retention in randomised trials provides insight into potential threats to internal and external study validity, and may inform the development of interventions to increase retention in future trials. Given a paucity of existing research in the field, a study was conducted to explore factors associated with retention in a smoking intervention trial involving persons with a mental illness, considering demographic and smoking characteristics, treatment condition and engagement in prior follow-up assessments.

**Method:**

A descriptive study was undertaken using data derived from a RCT of a smoking cessation intervention initiated in four adult psychiatric inpatient units in New South Wales (NSW), Australia. Retention assessment was undertaken at 1, 6 and 12-months post-discharge. A Generalised Linear Mixed Model was adopted to explore associations between retention at any follow up time point and demographic and smoking characteristics. Chi square analyses explored the association between retention at all follow up time points and treatment condition, and binary logistic regression analyses assessed for relationships between retention at 12-month follow up and engagement in prior follow up assessments.

**Results:**

Retention rates were 63, 56 and 60% at the 1, 6 and 12-month assessments, respectively. No association was found between retention at any follow-up time point and 13 of 15 demographic and smoking characteristics. Younger participants and those who identified to be Aboriginal and/or Torres Strait Islander were more likely to be retained (both *ps* > 0.05). Retention rates did not vary according to treatment condition at any follow-up time point. Participants who completed a prior assessment were more likely to complete the 12 month assessment (both prior assessments: *OR* 10.7, *p* < 0.001; 6 month assessment: *OR* 6.01, *p* < 0.001; and 1 month assessment: *OR* 1.8, *p* = 0.002).

**Conclusion:**

The underrepresentation of younger participants and those identifying to be Aboriginal and/or Torres Strait Islander may limit the generalisability of findings. Findings suggest that inclusion of multiple contacts during a trial follow up period may increase retention at the final assessment. Interventions to improve retention, overall and for those sub-groups less likely to be retained, in smoking trials involving persons with a mental illness are needed. Further assessment of sample characteristics, and also trial design factors, associated with retention in this field is warranted.

## Background

Tobacco smoking remains a leading cause of preventable morbidity and mortality worldwide [1]. In high income countries [2, 3] including Australia [4, 5], the prevalence of smoking remains disproportionately high among some population groups such as those with a diagnosed mental illness [6–8], where the prevalence has been estimated between 36 and 67% [9–11]. More rigorous intervention research tailored to smokers with a mental illness has been recommended to address the associated inequitable health burden [12, 13].

Low rates of participant retention at trial end-points or differential retention rates between treatment conditions can compromise the internal (inference that the intervention alone caused changes to outcome, through minimisation of potential confounding variables) and external (generalisability) validity and reduce the statistical power of controlled trials [14, 15]. As a result, the Consolidated Standards of Reporting Trials (CONSORT) statement [16] specifies that trials report retention rates overall and by each treatment condition separately. Given the risk of bias in trial results due to inadequate or differential participant retention, the development of novel approaches to increase retention rates has been identified as a priority for trial methods research [17].

Examining differences in the characteristics of participants retained and not retained has been suggested to provide insight into the validity of trial outcomes [18]. In the case of smoking cessation trials generally, differences in smoking-related behaviours and characteristics (such as motivation to quit or nicotine dependence) between retained and not-retained participants has the potential to confound trial results [15]. Given this, describing relationships between such participant characteristics and trial retention rates is recommended to aid the interpretation of reported trial findings [19]. Further, describing the relationships between participant retention and trial design elements may inform the development of interventions to increase retention rates in smoking trials [17, 20].

Among adult smokers generally, systematic review evidence suggests a variable relationship between participant retention and demographic characteristics such as age, gender and socio-economic status [21]; and this reflects findings from broader behavioural research [22]. In terms of smoking characteristics, lower levels of nicotine dependence [21, 23]; higher intention [21, 24], self-efficacy [21, 25] and motivation [21] to quit; and consumption of fewer cigarettes per day [21, 26] have been associated with participant retention.

Three trials undertaken in the USA have reported on the demographic and smoking characteristics associated with retention in smoking intervention trials involving persons with a mental illness [26–28]. Among smokers residing in the community with either current or past depression, gender, age, educational attainment and socioeconomic status were found to be unrelated to the stage that ‘drop out’ occurred (early, late and treatment completers) [26]; whereas less severe current depressive symptomology and consumption of fewer cigarettes per day were associated with ‘drop out’ stage [26]. In a trial involving psychiatric inpatients, participants without a co-morbid alcohol use disorder and those who earned a higher income were more likely to be retained at 3-months follow-up [27]. No smoking characteristics were found to be associated with retention [27]. In a further trial involving psychiatric inpatients, only readiness to quit at baseline was positively associated with retention [28]. No Australian research has examined demographic and smoking characteristics associated with retention in smoking trials involving persons with a mental illness.

Given the limited previous research, a study was conducted to explore factors associated with retention in a smoking intervention trial involving persons with a mental illness in Australia, considering demographic and smoking characteristics, treatment condition and engagement in prior follow-up assessments.

## Methods

### Design and setting

A descriptive study was undertaken using data derived from a two-arm, parallel group randomised controlled trial (RCT) of a smoking cessation intervention initiated in four adult psychiatric inpatient units in New South Wales (NSW), Australia and continued for 4 months post-discharge. Randomisation was carried out separately by unit and stratified by diagnosis (psychotic/non-psychotic) using a 1:1 allocation ratio. Participants allocated to the control condition received care as usual for smoking in the inpatient setting and following discharge. Those in the intervention condition, during the inpatient stay, received a brief motivational interview and written self-help material for smoking cessation; and following discharge, 16 weeks of telephone counselling and 12 weeks of fully subsidised nicotine replacement therapy. Follow-up assessments were undertaken at 1, 6 and 12 months post-discharge. The methods and outcomes of the trial have been previously reported [29, 30].

### Sample and recruitment procedure

Between October 2012 and April 2014, research staff liaised with nurse unit managers daily to identify new patients sufficiently clinically stable to be approached and assessed for study eligibility. Research staff were independent of the hospitals, received standardised training in mental illness and its impacts, and had completed or were in the process of completing an undergraduate degree in a health related area. To be eligible, patients: smoked any number of cigarettes in the month prior to hospital admission; were18 years of age or above; could provide informed consent; and had a current telephone number. Smokers did not have to be motivated to quit to participate in the trial. Eligible and consenting patients completed a baseline interview and were then randomly allocated to either a control or intervention condition (see [29] for the trial protocol).

### Retention strategies

The trial adopted several strategies that have been identified to increase retention in smoking cessation and other research fields, including: provision of reminders for upcoming assessments (via text or postal letter) [20, 31, 32]; reimbursement with a $20 supermarket voucher upon completion of each follow-up assessment [31, 33]; multiple (10) attempts to contact participants at each follow up point; collection of multiple contact details (landline and mobile phone numbers, email) for the participant, and the name and contact details of an elected contact person [20, 32, 33] and of their general practitioner (if applicable) [20, 32, 33].

### Data collection procedure

Participant retention was recorded by project staff conducting the computer-assisted telephone interview (CATI) at 1, 6 and 12 months post discharge (November 2012 to June 2015). Participants were called at each time point, irrespective of their completion status at a prior assessment. CATIs were the sole mode of follow-up data collection for the trial.

Participant characteristics of smoking and demographic data were collected for all participants via face-to-face interview during the inpatient stay. Other clinical and demographic information was obtained via the patient electronic medical record system at discharge.

### Measures

#### Participant retention

Retention was measured at the 1, 6 and 12 month post -discharge follow-up assessment. Participants were considered ‘retained’ if they fully or partially (answered at least questions assessing current smoking behaviour) completed the follow-up assessment.

#### Demographic information

The following demographic information was obtained from via face-to-face interview during the inpatient stay: employment details (full time, part time, household duties, student, unemployed/ other), education (primary school, third year of high school, school certificate (fourth year high school), Higher School Certificate (HSC; sixth year high school), tertiary qualification not obtained from a university, bachelor degree, post graduate degree) and living circumstances (on own, with others).

The following data were collected from the patient electronic medical record system at the time of discharge: primary mental health diagnosis (schizophrenia and related psychoses, anxiety and stress related disorders, mood disorders, substance- related disorders, personality and other disorders), age, gender, relationship status (single, married/de facto, separated/divorced, widowed, did not state/inadequately described),Aboriginal and/or Torres Strait Islander status (Aboriginal and/or Torres Strait Islander, neither, did not state), legal status at admission (voluntary, involuntary), and length of stay (number of days between admission and discharge).

#### Smoking characteristics

Smoking characteristics collected via face-to-face interview in the inpatient setting included: smoking status (daily smoker, weekly smoker, irregular smoker [smoked cigarettes less than weekly), cigarettes per day, level of nicotine dependence (Fagerstrom Test for Nicotine Dependence [FTND]) [34], ‘readiness’ to quit smoking (Readiness to Quit Smoking Questionnaire [35]), and number of quit attempts in the past 6 months (0, ≥1).

### Variable transformation

The following variables were categorised to two levels for association analyses: diagnosis (psychosis, non-psychosis), Aboriginal and Torres Strait Islander status (Aboriginal and/or Torres Strait Islander, neither Aboriginal nor Torres Strait Islander), employment status (paid workforce, unpaid workforce), educational attainment (HSC or lower, tertiary), relationship status (currently partnered, not-partnered/single), smoking status (daily smoker, weekly/ irregular smoker), nicotine dependence (low-moderate [FTND score ≤ 5], high [FTND score ≥ 6]) [36], and readiness to quit (pre-contemplative, contemplative or a more progressed stage). Age was reduced to four levels: (18–25, 26–35, 36–50, 51+) [37].

### Analyses

Analyses were conducted using IBM SPSS Statistics 22 [38].

Descriptive statistics were used to report on overall retention rates, and according to condition of allocation. McNemar tests assessed for differences in retention rate at the 1, 6 and 12 month follow-up assessments.

Due to multiple pairwise comparisons in this study, a priori all analyses were conducted with a type I error of ∝= 0.01.

#### Demographics and smoking characteristics associated with retention at any follow-up time point

A generalised linear mixed model (GLMM) was adopted to model participant retention (dependent variable) at any follow-up assessment, and explore potential associations between retention and demographic and smoking characteristics. A compound symmetry residual covariance structure was used to model correlation associated with repeated time measurements. All measures included in Table 1 were considered as independent variables in the GLMM. Characteristics associated with retention at any follow-up time point were determined via significant main effects. Allocation was entered and retained in the model for control purposes.

**Table 1:** Baseline participant demographic information and smoking characteristics by treatment condition

	Control *(n* = 367)	Intervention *(n* = 373)	Total *(N* = 740)
Age^a^	38.30 (12.01)	39.08 (11.96)	38.69 (11.99)
Age initiated smoking^a^	15.45 (4.40)	15.61 (4.83)	15.53 (4.62)
Length of stay^a^ (days)	13.36 (15.92)	15.11 (18.78)	14.24 (17.43)
Cigarettes smoked per day^a^	21.02 (13.19)	21.81 (14.49)	21.42 (13.86)
Gender^b^ (male)	224 (61%)	228 (61%)	452 (61%)
Cultural background^b^ (ATSI)	48 (13%)	23 (14%)	101 (14%)
Diagnosis^b^ (psychosis)	82 (22%)	84 (23%)	166 (22%)
Legal status^b^ (involuntary)	167 (46%)	179 (48%)	346 (47%)
Relationship status^b^ (single)	279 (76%)	306 (82%)	585 (79%)
Living circumstances^b^ (own)	103 (28%)	116 (31%)	219 (30%)
Highest education level^b^ (HSC or lower)	258 (70%)	292 (78%)	550 (74%)
Employment status^b^ (unpaid workforce)	267 (73%)	277 (74%)	544 (74%)
Type of smoker^b^ (daily)	342 (93%)	348 (93%)	690 (93%)
Readiness to quit^b^ (pre-contemplation)	200 (55%)	207 (56%)	407 (55%)
Number of quit attempts^b^ (at least one)	107 (29%)	121 (32%)	228 (31%)
Nicotine dependence^b^ (high)	189 (52%)	192 (52%)	381 (52%)

^a^Mean (SD); ^b^Number (%); ATSI: Identify to be Aboriginal and/or Torres Strait Islander origin; HSC: Higher School Certificate. Note. Table 1 presents summarised clinical, demographic and smoking characteristic data. For non-summarised data, please see Table 1 in Metse et al. [30]

#### Association between retention and treatment condition at all follow-up time points

Chi square analyses assessed potential variations in retention rate according to treatment condition, at each follow-up assessment.

#### Association between retention at 1 and 6 month follow-up assessments, and retention at 12 months

Binary logistic regression was used to explore associations between retention at 1 and 6 month assessments, with retention at 12 months. Interactions between retention status at 1 and 6 month assessments and condition of allocation were also entered into the model.

## Results

### Sample

**Fig. 1:**
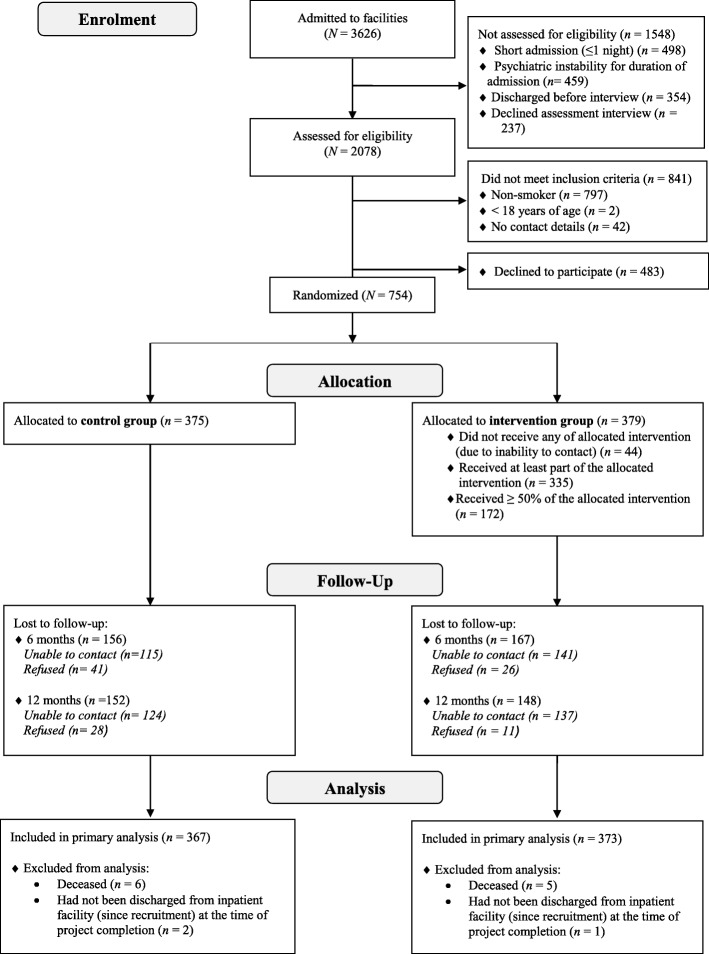
Participant flow diagram

Three thousand six hundred and twenty-six patients were admitted to the psychiatric inpatient facilities during the recruitment period; 2078 were assessed for eligibility, and 61% (*N* = 754) of eligible smokers were recruited into the smoking cessation intervention trial (Fig. 1). Fourteen participants were excluded from this study due to being deceased (*n* = 11) or not discharged from hospital (*n* = 3) at project completion - leaving 367 and 373 in the control and intervention condition, respectively. Participant demographic information and smoking characteristics, by treat condition, are summarised in Table 1.

### Participant retention

The rate of participant retention did not vary between the 1 and 12 month post-discharge assessments (63% versus 60%, *p* = 0.13) or the 6 and 12 month assessments (56% versus 60%, *p* = 0.112). However, significantly more participants were retained at the 1 month compared to 6 month follow-up assessment (63% versus 56%, *p* = 0.001).

### Demographics and smoking characteristics associated with retention at any follow-up time point

The large majority (13/15) of demographic and smoking characteristics considered were not associated with retention at any follow-up time point (*p*s > 0.05). Retention was found to be significantly associated with age and Aboriginal and Torres Strait Islander status. The likelihood of retention was lower for those aged 18–25 (*OR* 0.83, 95% CI 0.75 to 0.91, *p* < 0.001), 26–35 (*OR* 0.88, 95% CI 0.80 to 0.95, *p* = 0.003) and 35–50 (*OR* 0.88, 95% CI 0.81 to 0.95, *p* = 0.002) compared to those aged 51 and over; and for those who identified to be Aboriginal and/or Torres Strait Islander, relative to those who did not (*OR* 0.88, 95% CI 0.81 to 0.95, *p* = 0.002).

### Association between retention and treatment condition at all follow-up time points

There was no evidence of differential retention between treatment conditions at any follow-up assessment (Table 2).

**Table 2:** Participant retention at 1, 6 and 12 months post-discharge by condition

Follow-up Assessment	Control (*n* = 367)	Intervention (*n* = 373)	*p*
1 month	232 (63%)	233 (62%)	0.833
6 month	211 (58%)	206 (55%)	0.534
12 month	215 (59%)	225 (60%)	0.630

### Association between retention at 1 and 6 month follow-up assessments, and retention at 12 months

The odds of retention at the 12 month post-discharge assessment were 1.8 (95% CI 1.25 to 2.59, *p* = 0.002) times higher for those retained at 1 month, compared to those who were not. The odds of retention at the 12 month post-discharge assessment were 6.01 (95% CI 4.20 to 8.60, *p* < 0.001) times higher for those retained at 6-months, relative to those who were not. Participants retained at either the 1 or the 6 month post-discharge assessment were 2.4 times more likely than those who had completed no previous assessments to be retained at 12 months (95% CI 1.58 to 3.61, *p* < 0.001). While those who completed both prior assessments were 10.7 times more likely to be retained at 12 months (95% CI 7.12 to 16.11, *p* < 0.001) than those who had completed neither assessment. Likelihood of retention contingent on previous assessment completion status did not vary according to allocation condition.

Of the 201 participants not retained at either the 1 or 6 month assessment, 30% (*n* = 60) were retained at 12 months. Similarly, 51% (99/196) of participants who completed only one of the prior assessments completed the 12 months assessment.

## Discussion

Low rates of participant retention or differential retention can compromise the internal and external validity of controlled trials [14, 15]. Examining differences in the characteristics of participants retained versus those who are not has been suggested to provide insight into threats to trial validity [18], and may inform interventions to increase retention in future trials. This study adds to a very limited literature [26–28] exploring factors associated with retention of persons with a mental illness in smoking cessation trials. While 13 of 15 the demographic and smoking characteristics considered were not associated with retention, it was found that younger participants and those who identified to be Aboriginal and/or Torres Strait Islander were less likely to be retained, suggesting they are underrepresented which could limit the generalisability of findings. No smoking characteristics were found to be associated with retention, and there was no evidence of differential retention between treatment conditions. This study also assessed the association between completion of prior follow-up assessments and retention at the trial end point, with results suggesting a significant positive relationship. Findings indicate additional approaches to improve retention in smoking cessation trials involving persons with a mental illness are needed, both overall and for sub-groups identified to be underrepresented in this study. The inclusion of multiple contacts across a trial follow up period may increase participant retention at the final assessment. Retrospective, systematic identification of sample and trial design factors associated with participant retention in smoking cessation trials involving persons with a mental illness is needed to inform the development of interventions to increase retention rates, tailored to the field.

The demographic characteristics found to be associated with retention in the current study were similar to those reported for other studies, both within and outside the field of smoking research. In terms of participant age, research across the health behaviour change field broadly has demonstrated that older participants are more likely to remain engaged in research trials [39–41], with such a trend also evident in smoking intervention research [21]. To ensure younger persons with a mental illness are represented in trials of smoking cessation interventions, approaches to increase retention are needed. For example, this subgroup has been shown to prefer a more personal mode of follow-up (face-to-face rather than online/telephone surveys; [42]). Choice of participation mode has also been shown to increase retention [43], therefore, offerring the options of more and less personal follow-up modes may be an intervention to increase retention of young persons in smoking cessation intervention trials. Further research is needed to determine the impact of such an approach.

The finding that participants who identified to be of Aboriginal and/or Torres Strait Islander origin had a lesser likelihood of being retained is congruent with that of previous health behaviour research involving indigenous minority groups [44–46], including those focussed on smoking cessation interventions [21]. Limited research has described possible strategies for increasing retention of such participants in research trials [33]. Cultural tailoring is one approach that may improve engagement and in turn, retention [47, 48]. Cultural tailoring involves the consideration of a target population’s ethnic/cultural characteristics, experiences, norms, values, behavioural patterns and beliefs, as well as relevant historical, environmental, and social forces when designing, delivering, and evaluating a health behaviour trial [49]. Assessment of the impact on retention of culturally tailoring interventions to reduce smoking among persons with a mental illness who identify as Aboriginal or Torres Strait Islander is needed [47, 48].

No smoking characteristics were associated with retention in the current study, suggesting the trial outcomes were not confounded [15]. These findings are similar to those of a US study involving smokers admitted to inpatient psychiatry, where smoking characteristics were also found to be unrelated to retention [27].

The finding that participation in prior follow-up assessments increased the likelihood of retention at the final assessment is supportive of prior research suggesting maintenance of contact with participants across the follow-up period facilitates retention [33, 50, 51]. Such results suggest that intermediate follow-up assessments not only provide extended behaviour-change data, but serve as a trial design strategy to improve retention at the primary follow-up point.

In terms of existing evidence on strategies to increase retention in trials, a Cochrane systematic review and meta-analysis involving 38 trials across a spectrum of disease areas found a significant effect of monetary incentives, tracking of postal questionnaires and open trial designs [31]. The generalisability of findings across follow-up methods, however, is somewhat limited as 34 of 38 included trials assessed response to postal or electronic questionnaires. Further, the broad inclusion criteria for the review and heterogeneity of included studies limited capacity to consider findings by population group or disease area. Given retention rates are likely impacted by unique factors within population groups and disease areas [51], and the adoption of universal strategies to increase retention often have only a modest impact, as is the case for the current study; targeted research is required to achieve meaningful increases in retention rates in specific research fields.

The strengths of this study include its conduct with a large and diverse sample of smokers with a mental illness and a 12-monthfollow-up period. However, participants were recruited across four psychiatric hospitals in one regional local health district in NSW, Australia and therefore findings may not be generalisable to samples recruited from non-acute facilities, or for those residing in capital cities or rural and remote areas. In addition, full consideration of all factors that may have an impact on retention, such as those related to the trial design/delivery and measures of participant motivation and characteristics of recruiting staff [52], was outside the scope of this paper and hence these may represent confounding variables. Assessing factors associated with retention for the intervention and control conditions separately was also outside the scope of this paper. Finally, interviews were not conducted with those who withdrew consent to participate, and as a result reasons for such could not be included in the regression model. Nevertheless, the results of this study provide an indication of the characteristics of persons with a mental illness, in an Australian context, who may be less likely to be retained in smoking trials, irrespective of the reason for non-completion of follow-up assessments.

## Conclusions

The underrepresentation of younger participants and those identifying to be of Aboriginal and/or Torres Strait Islander origin may limit the generalisability of findings from the overarching RCT. Approaches to maximising retention of these groups in future smoking trials involving persons with a mental illness are needed. Findings suggested that inclusion of multiple contacts during a trial follow up period may increase retention at the final assessment.

To increase retention rates in smoking cessation trials involving persons with a mental illness, further research of high methodological rigour, such as a systematic review, is needed to identify sample and trial design factors associated with participant retention. Such research would then inform the development of interventions/strategies to maximise retention, tailored to the field.

## Abbreviations

CI: Confidence interval
CONSORT: Consolidated Standards of Reporting Trials
GLMM: Generalised linear mixed model
NSW: New South Wales
RCT: Randomised controlled trial
RR: Relative risk
